# Spatial Ecology of Livestock Protection Dogs, Sheep, and Pampas Foxes in Agroecosystem of Central Argentina

**DOI:** 10.3390/ani16020180

**Published:** 2026-01-08

**Authors:** Sabrina Daniela Martínez, Mauro Lucherini, Nicolás Carmelo Caruso, Emma Beatriz Casanave, Estela Maris Luengos Vidal

**Affiliations:** Grupo de Ecología Comportamental de Mamíferos (GECM), Departamento de Biología, Bioquímica y Farmacia, Instituto de Ciencias Biológicas y Biomédicas del Sur (INBIOSUR), Consejo Nacional de Investigaciones Científicas y Técnicas (CONICET), Universidad Nacional del Sur (UNS), Bahía Blanca B8000, Buenos Aires, Argentina; sabrina.martinez@uns.edu.ar (S.D.M.); lucherinima@yahoo.com (M.L.); nccaruso@gmail.com (N.C.C.); ebcasanave@gmail.com (E.B.C.)

**Keywords:** human–wildlife conflict mitigation, non-lethal tool, sheep, livestock protection dog, *Lycalopex gymnocercus*, agroecosystem

## Abstract

Livestock protection dogs (LPDs) are specifically bred to protect livestock, such as sheep, from predators. These dogs’ primary defense mechanisms include directional actions that deter predators but no attack. Little information is available on the influence of LPDs on the spatial ecology of predators. We analyzed interactions between an LPD, sheep, and Pampas foxes (*Lycalopex gymnocercus*, a main predator of lambs) in a ranch located in central Argentina. Between 2018 and 2021, we deployed GPS collars on an LPD and 2 ewes from a flock of 300 sheep and VHF collars on 12 live-trapped Pampas foxes. The home ranges (HRs) of the LPD and the ewes overlapped largely, especially during the lambing period, with the LPD performing minimal excursions outside the area used by the sheep flock. The LPD appeared to effectively reduce predation on lambs. Foxes exhibited a large HR (average 6.42 km^2^), with considerable intraspecific overlap. The overlaps between the HRs of the foxes and the LPD were variable (range = 0–98%), but their respective core areas never overlapped, and the minimum distance between the core area centers was 950 m. This study highlights the effectiveness of LPDs at reducing predation while enabling the permanence of carnivorous predators in the ecosystem.

## Simple Summary

The effectiveness of livestock protection dogs (LPDs) in reducing livestock predation has been widely documented. They are considered a valuable non-lethal tool for mitigating predation because, in addition to protecting livestock, they contribute to the conservation of carnivorous species and maintaining ecological balance. However, there is little empirical evidence on predator behavior in the presence of LPDs. This study aimed to investigate the interactions between LPDs, sheep, and Pampas foxes, the main predator of lambs, in the study area of central Argentina. To this end, GPS collars were placed on dogs and sheep, while captured foxes were monitored with VHF collars. The results showed that LPDs effectively reduced lamb predation, although they did not completely exclude foxes from the area; therefore, temporary expulsion responses to encounters occurred on occasion. Foxes displayed larger home ranges than reported elsewhere, which remained stable throughout the year, while maintaining their distance from the LPDs and engaging in direct but non-lethal encounters. Overall, this study underscores the role of LPDs as an effective management tool, minimizing livestock losses while allowing carnivores to persist in the ecosystem.

## 1. Introduction

Dogs (*Canis familiaris*) have been associated with humans since prehistoric times for various tasks, particularly herding and protecting livestock from both carnivore predation and rustling, as recorded in ancient writings and art [1]. Selection for certain dog characteristics has enabled the development of different breeds, including those used as livestock protection dogs (LPDs) for non-lethal mitigation of carnivore predation. LPDs have been used as a tool for at least 6000 years in European countries [2,3,4,5]. LPDs not only reduce actual or perceived damage from livestock attacks but also increase predator tolerance, making them a valuable tool for the conservation of conflictive species. Despite these benefits, there are several potential ecological side effects of this tool [6].

The different strategies adopted by LPDs to deter predators can vary depending on both the breed of the LPD and the predator species. LPDs could be a threat to both native predators and prey species, potentially affecting wildlife almost as strongly as the use of lethal measures. However, little information is available on the effect of LPDs on wildlife and particularly on the predators they are expected to deter. LPDs have been reported to spatially exclude red foxes (*Vulpes vulpes*) [7] or modify predator behavior (interaction interference). In Australia, LPDs did not displace red foxes, but they did modify their behavior [8]. In recent years, the number of farmers adopting this measure to mitigate conflict between carnivores and sheep has increased considerably in Argentina [9]. However, although the results in some cases are promising, the differences between the environments in which LPDs are used, predator communities, and sizes and management strategies of livestock on each farm have led to frequent questioning of the efficiency and usefulness of LPDs. In this study, we aimed to understand the effects of LPDs—as a non-lethal predation mitigation tool—on wildlife, particularly on the spatial ecology of the Pampas fox (*Lycalopex gymnocercus*) in a sheep production context in southern Buenos Aires province, central Argentina. The Pampas fox is locally considered one of the carnivores that more frequently predates on sheep, especially lambs [10,11,12,13,14].

The Pampas fox is a medium-sized fox. It inhabits the southern part of South America, occupying almost all habitats [15,16]. It is a widespread species throughout its range, despite being persecuted due to conflicts with humans. Although it is a hunted species, it is very abundant both in protected areas and in anthropogenically modified environments [16,17,18]. Its diet is generalist, including fruits, insects, crabs, birds, native and exotic rodents, and the exotic European hare (*Lepus europaeus*) [19,20,21,22]. In farmlands, it also feeds on lambs or chickens, either by preying or scavenging. At the local level, the diet of Pampas foxes can be highly variable and may be influenced by human activities such as livestock farming, agriculture, and the presence of introduced species [17,23,24,25]. In central Argentina, the mating season is from September to December [26], and the young remain in the burrows under the care of both parents until approximately 6 months of age [27]. Interactions with feral or domestic dogs are infrequent, as domestic dogs are usually trained to hunt and kill them, and only one case of hybridization is known [28].

In this study, three predictions concerning the hypothesis that LPDs and sheep coexist with Pampas foxes are examined: (I) LPDs use essentially the same territory as the sheep they protect, although LPDs may range over larger areas to deter potential predators; (II) foxes alter their spatial behavior during the lambing period, as protection dogs create a fear-based territory due to their close proximity to sheep, forcing foxes to expand their range in search of food; and (III) foxes use the area occupied by sheep and the LPD only for occasional incursions or remain in the peripheral parts of the dog’s territory.

## 2. Materials and Methods

### 2.1. Study Area

The study was carried out at the Patagones Experimental Farm (PEF) (40.6472° S, 62.8842° W), a farm owned by the Ministry of Agrarian Development of the Province of Buenos Aires, located in the Patagones district, central Argentina. The farm covers 1212 ha, of which 59% consists of woodland and natural grasslands. The PEF lies within Argentina Espinal ecoregion, at the transition zone with Patagonian woodland and steppe. Natural vegetation includes dry scrub and xerophytic forests dominated by chañar (*Geoffroea decorticans*), rockrose (*Larrea divaricata*), and the endemic piquillín (*Condalia microphylla*), together with halophytic grasslands and cacti (*Opuntia* spp., *Cereus* spp.). The area has been heavily modified by farming expansion and transformed into a mosaic of grasslands, crops, and remnants of original vegetation [29]. At PEF, forest and grassland predominate, with smaller areas of shrubby grassland. The land is divided into paddocks enclosed by wire fences to control livestock movements, but fences do not prevent the entry of predators.

### 2.2. GPS Collar Placement

In September 2021, collars with CatLog2^®^ GPS receivers (Perthold Engineering LLC, Anderson, SC, USA) were placed on an LPD (a castrated adult male of the Maremmano–Abruzzese breed) and, consecutively over time, on 2 pregnant adult ewes from the flock of 316 mother ewes protected by the dog. It was assumed that the spatial behavior of each ewe was representative of the flock’s [30,31], and from this point onward, these data are considered to represent the sheep home range. The GPS unit included onboard memory and rechargeable batteries, and recorded one location every 5 min, 24 h a day.

### 2.3. LPD and Sheep Management

The LPD’s behavior had been visually monitored since its arrival at the farm (August 2017), and its performance in reducing predation was consistently evaluated as effective by farm staff. Sheep rotated among different paddocks throughout the year for grazing. The dog’s feeder was located within the grazing areas, and water was always available to the flock. During lambing, sheep were confined at night, since newborns are especially vulnerable to predation during the first 15 days after birth [32]. After parturition, ewes with lambs remained for 30 days in an open paddock near the farmhouse. Breeding combined artificial and natural insemination, with rams present in the field. Consequently, lambing extended along 45 days, with two peaks corresponding to each insemination type. Pregnancy was assessed by ultrasound to estimate birth rates. Occasional interactions between the guard dog and predators were documented through direct observation or audiovisual recordings. In addition, records of sheep losses and their causes, maintained by PEF staff, were used to evaluate the dog’s effectiveness.

### 2.4. Capture, Collaring, and Tracking of Pampas Foxes

Between 2018 and 2021, live-capture sessions targeting *Lycalopex gymnocercus* were conducted at PEF. Foxes were trapped with modified padded leg-hold traps (Victor Soft Catch^®^ 1 ½, Oneida Victor, Cleveland, OH, USA) [33]. Each captured individual was sexed, weighed, and measured for standard morphometrics. All of them were marked with individually coded ear tags and released at the capture site once fully recovered. A subset of healthy adult foxes was fitted with collars containing Wildlife Materials^®^ HLPM 3140 Very-High-Frequency (VHF) transmitters (Murphysboro, IL, USA). Collars weighed 140–160 g, which was <3% of the animal’s body mass in all cases [34].

Fox locations were obtained by ground or vehicle tracking at different times of day (every 6 h to obtain independent records). Locations were determined by triangulation using a three-element Yagi antenna and portable receivers (Wildlife Materials^®^ AVM^®^ LA 12-Q and TRX-16S). The operator’s position was georeferenced with a GPS unit, and azimuth relative to magnetic north was measured with a compass by identifying the strongest signal.

### 2.5. Data Processing and Analysis

GPS collar records were screened to remove erroneous data. For sheep, positions outside fenced areas or with speeds > 3 km/h were discarded [35]. For both species, outliers indicating unrealistic movements (<0.2% of the data) were also excluded. Triangulation-derived fox locations were processed using the SIGLOC package 0.0.4 [36] in R 4.2 [37], filtering out those with a high error margin, e.g., positions extremely far from the established area for each fox.

Home ranges (HRs) of foxes, the LGD, and sheep were estimated using the kernel method [38] implemented in the adehabitatHR package v.1.4 [39]. This method estimates both the probability of presence and the intensity of space use, while being less sensitive to extreme values [40].

For the LGD, space use was analyzed in relation to both the radio-tagged sheep within the flock and foxes. Comparisons were made using 95% kernel HR, a scale widely recommended for size and overlap comparisons [41,42,43], in QGIS^®^ 3.10 [44]. For this analysis, we separated the lambing period (45 days, September–November), considered a critical stage for ewes and lambs [11], from the rest of the year. Fox data from the 2019 and 2021 lambing periods were included. Due to the high overlap between the LPD and ewe HRs and the fact that each year, during lambing, the flock remains in proximity to the LPD in the same paddocks, the LPD and ewe data obtained during the 2021 lambing were used to extrapolate and compare them with the HRs of foxes monitored in previous years.

HR overlap was quantified with the Bhattacharyya coefficient (0 = no overlap, 1 = complete overlap; [45]). Additionally, the 30% kernel HR was estimated to represent the core area of intensive use [46,47,48]. Finally, the distances between the arithmetic centers of 30% HRs were also calculated, as a further measure of spatial segregation [31,49].

## 3. Results

### 3.1. Spatial and Temporal Monitoring of the LPD, Sheep and Pampas Foxes

#### 3.1.1. Recording of Lamb Losses and LPD–Predator Interactions

At the PEF, a comparison between 2015–2016 and 2018–2020–2021 indicated a 7% reduction in lambing losses (measured as born lambs that survived to the age of one month). During data collection, no cases of direct predation on lambs by foxes were recorded. Four direct interactions were observed between the LPD and foxes, consisting of scaring and chasing behavior, but no physical attacks.

#### 3.1.2. Sampling Effort

In 2021, GPS collars recorded 89,802 positions for the LPD and 48,108 positions for the ewe. Lambing occurred from 31 August to 15 November. The LPD GPS functioned from 23 September 2021 to 6 March 2022 (approximately 6 months), after which the collar was lost. The ewe’s GPS operated from 22 September to 16 December 2021 (4 months).

#### 3.1.3. Home Range Size

The ewe’s HR sizes within and outside the lambing period were very similar; the absolute differences in square kilometers were 0.05, 0.01, and 0.09 for 30, 95, and 99% HR estimates, respectively (Table 1). For LPD, the 95% HR was larger outside the lambing period (0.29 km^2^), and this difference was even greater for the 99% HR (0.44 km^2^).

Twelve live trapping campaigns were carried out, totaling 3476 trap-nights (mean: 289.6 trap-nights per campaign). Sixteen Pampas foxes were captured, yielding a capture rate of 0.46 foxes/100 trap-nights. No recaptures were recorded. We released three pups due to small size, and a fourth individual due to the lack of available collars. The remaining 12 individuals were fitted with VHF collars.

Only 7 of the 12 collared foxes (2 females and 5 males) were monitored between 5 and 15 months (median: 8 months). The rest were followed for ≤2 months, primarily due to equipment failure, and excluded from the analysis. Between 2018 and 2021, a total of 576 locations were obtained (range: 46–118 locations per individual). The data sample was sufficient to compare HR sizes with non-lambing periods only for the 2019 and 2021 lambing periods.

The mean Pampas fox HR size was 6.42 km^2^ (range: 2.82–9.71 km^2^) using the 95% kernel, with large inter-individual variation. We found no differences between sexes in HR sizes (95% kernel), with females (*n* = 2) averaging 6.72 km^2^ and males (*n* = 5) averaging 6.3 km^2^. During lambing, data were obtained from 5 individuals who had a mean HR size (95% kernel) of 6.24 km^2^ (range: 2.59–8.09 km^2^, Table 2). Seven individuals were monitored outside of lambing and showed an average HR size of 6.24 km^2^ (range: 3.02–10.8 km^2^), showing no differences between periods.

#### 3.1.4. Monthly Home Ranges

The ewes’ HRs showed a stable size pattern across months, with the exception of September, when lambing began. During this month, movements were more limited because sheep was forced to spend the nights in a pen and the days near the house; the HRs at 30%, 95%, and 99% were smaller in September than in the following months. In contrast, the LPD exhibited more pronounced monthly variations, with a notable decrease in February (both 95% and 99% HRs, Table 3).

#### 3.1.5. Home Range Overlap

**Figure 1:**
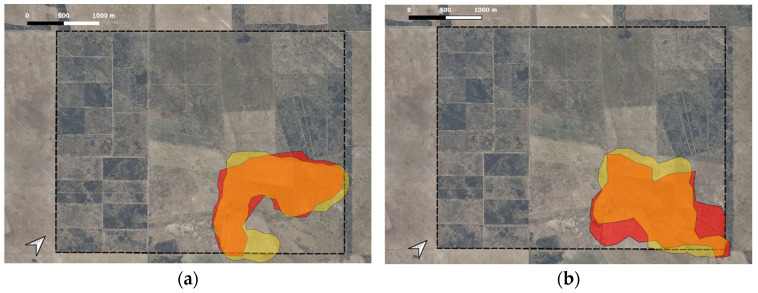
Overlaps in HRs between the GPS-collared LGD and ewe during lambing (**a**) and outside of lambing (**b**). The red area represents the HR of the LPD, the yellow area the HR of the ewe, and the orange area the overlap between the areas of both individuals. The box with dotted lines represents the limit of the Patagones Experimental Farm, central Argentina.

The ewes’ HR overlapped with that of the LPD by 78.1% during lambing and 79.3% outside this period. Conversely, the LPD’s HR was occupied by the ewe by 89.9% during lambing and 75% outside of lambing (Figure 1). The Bhattacharyya coefficient had a value of 0.86 and 0.76, respectively.

#### 3.1.6. Spatial Dynamics Between Livestock Protection Dogs, Sheep, and Pampas Foxes

Only 4 of the 7 radiocollared foxes shared their territories with those of the LPD and sheep outside the lambing period (Table 4). Two male foxes used almost the entire HR of both the LPD and sheep (>90%). However, the portion of the LPD’s HR overlapping with these two foxes was relatively small, ranging between 20% and 30%. Similar values were recorded for the sheep (Table 4). For the remaining 5 foxes overlapping with the LPD and sheep, the percentage overlaps in HRs were either zero or very low in both directions (0–20.9%). The Bhattacharyya coefficients were intermediate for foxes with the greatest overlap with the LPD and sheep, while they were zero for the other foxes (Table 5).

During the lambing period, only three foxes shared any overlap between their HRs and those of the LPD and sheep. Among them, a male (M5) maintained similarly ample overlap percentages compared to values outside the lambing period. The only female showing HR overlap with the LPD and sheep (F2) increased this overlap, reaching almost 50% of the LPD’s HR (compared to only 11% outside lambing, Table 6). Conversely, the portion of the LPD’s HR overlapping with F2 increased from 4% outside lambing to 9.6% (Table 6). M3 went from having no overlap outside lambing to overlapping similarly to F2 (10% of the LPD’s HR), while only 2.7% of his own HR overlapped with the LPD (Table 6).

**Figure 2:**
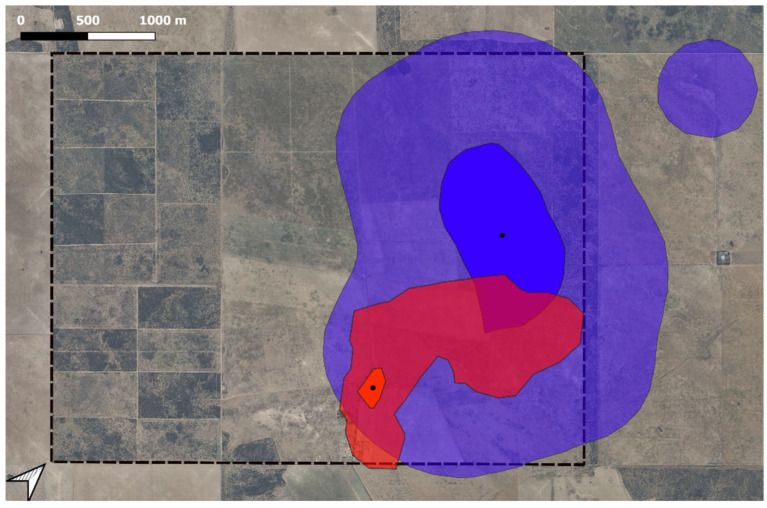
Overlap between the HRs of a male Pampas fox and a livestock protection dog (LPD) during the lambing period. The red area represents the HR of the LPD and the blue area represents the HR of the male Pampas fox; in both HRs, the larger, transparent areas represent 95% HR, the more solid-colored areas the 30% HR, and the black dots the HR centers. The box with dotted lines represents the limit of the Patagones Experimental Farm, central Argentina.

The Bhattacharyya coefficients (Table 7) confirm that the highest overlap occurred between the male fox (M5) and both the LPD and sheep (Figure 2). The coefficient for F2 and the LPD and sheep was 0.15, indicating lower overlap.

Comparing the arithmetic centers of the HRs of the five foxes with data in both periods to the centers of the HRs of the LPD and sheep, we found that two individuals shifted their centers away during lambing (66 m and 135.8 m), while the remaining foxes had closer centers (235.6 m, 247.6 m, and 539.6 m) (Table 8). The variations in distances were generally low, representing 2% to 36% of the total distances between HR centers. The distance between the HR centers of the sheep and the LPD was smaller outside the lambing period than during lambing.

## 4. Discussion

This study integrated ecological, spatial, and behavioral data of an LPD, the sheep it guards, and the Pampas fox, an important predator in sheep production systems in central Argentina. The results indicate that the LPD exhibited strong spatial fidelity to the flock it protected during both lambing and non-lambing periods, thereby lending support to the first hypothesis, with its home range (HR) largely and consistently overlapping that of the sheep. This association reflects effective imprinting, likely resulting from successful early socialization of the dog with the livestock, which is considered a critical step in the process to properly train an LPD [50]. Notably, the LPD’s HR size was similar to that of the sheep, despite the dog’s greater mobility and capacity to cross physical barriers such as fences. The low frequency of excursions outside the flock area suggests that the reliable access to food and water almost entirely eliminated the need for external resources by the LPD, indicating both functional efficiency and potential animal welfare benefits [51]. These results contrast in part with those reported by van Bommel and Johnson (2015) [51] in a production system with dingoes (*Canis dingo*) in Australia, where LPDs ranged beyond the immediate vicinity of the livestock and defended a larger territory, despite having resources such as water and food covered.

Sheep spatial behavior was consistent with patterns observed in other extensive ranching systems, showing homogeneous use of paddocks and equitable grazing distribution, a pattern that was likely influenced by rotational paddock management and flock social structure [52]. Complete utilization of available space may also be associated with the presence of the LPD, which reduces perceived predation risk [8,53].

In contrast, the Pampas fox exhibited more flexible and variable spatial behavior. HR sizes obtained in this study averaged 6.42 km^2^ (95% kernel) and were larger than those reported in other sites of this area, confirming the great ecological plasticity of this canid [54]. The study area comprises pasturelands with low food availability and scrublands that offer comparatively higher resource abundance. Consequently, foxes likely maintain home ranges large enough to encompass scrubland patches, resulting in extensive sectors of low-use pasture within their overall range. The variability in HR sizes may be driven by the dispersal of food resources, human disturbance, and intraspecific territorial dynamics [40,43]. The minimal differences in HR size and habitat use we found between lambing and non-lambing periods support the idea that the temporary presence of lambs does not constitute a sufficiently strong resource to alter Pampas fox spatial organization, in contrast to our second prediction.

Although substantial interspecific spatial HR overlap occurred between some Pampas foxes, the LPD, and sheep, core areas remained largely distinct. Foxes never used the LPD’s central area of intensive use, suggesting active avoidance behavior. This phenomenon has been documented in other predator–LPD interactions [55,56], where the dog’s presence generated an exclusion effect through perceived risk rather than territorial defense behavior (sensu van Bommel & Johnson, 2014 [50]). Furthermore, direct observations and video recordings showed the LPD intercepting, vocalizing at, and chasing foxes without attacking, demonstrating typical non-lethal defense behavior, as observed with other LPD breeds [57].

The results provide partial support for the third hypothesis. Although there is some overlap between the home range (HR) of the livestock-protecting dog (LPD) and that of foxes, this overlap is confined to the peripheral areas. The core areas of use do not overlap and remain spatially segregated. The recorded overlap suggests that exclusion is not absolute but temporary, with instances of direct pursuit occurring, consistent with the observations reported by van Bommel & Johnson (2024) [53].

Probably, coexistence between the LPD and Pampas foxes was also made possible by segregation in activity: in this area, the LPD was primarily diurnal [58], whereas foxes were crepuscular–nocturnal [59]. In sites of the same area with high human activity and no LPD, Pampas foxes increased nocturnal activity, probably in response to hunting pressure, consistent with observations elsewhere [59].

From a conservation and management perspective, the use of LPDs produced positive ecological and productive impacts. Reduced predation losses can minimize the need for lethal control measures such as hunting or poisoning that often have negative consequences for biodiversity [60]. In areas without LPDs, hunting pressure on carnivores can trigger compensatory population responses that perpetuate human–wildlife conflict [61]. At the PEF, fox live trapping success rates were three times higher than in areas without LPDs and hunting activity [62], possibly reflecting an indirect ecological benefit: in the absence of lethal measures, predator populations can remain stable and even serve as sources for neighboring, less hospitable areas, favoring a regional source–sink dynamic.

Producers using LPDs also reported benefits, including improved wool quality, calmer flocks, and easier sheep management [63]. Economically, LPDs reduce livestock losses, although acquisition and maintenance costs limit accessibility for some producers [64]. Failures tend to be associated with inadequate LPD implementation, particularly when the dog–flock bond is weak, underscoring the need for training programs, technical monitoring, and dissemination of best practices [65].

## 5. Conclusions

Overall, our findings reinforce that well-selected and properly raised and managed LPDs are tools for the non-lethal mitigation of livestock–carnivore conflicts that combine effectiveness, ecologically viability, and societal approval. However, integrated, coordinated approaches at a regional scale are essential to avoid conflicting strategies, such as the use of LPDs on one farm and poison use on a neighboring property—which could undermine effectiveness and disrupt predator–prey dynamics—and ensure ecological, economic, and social sustainability in these production systems. Future studies should assess LPD effects on other predators (e.g., pumas *Puma concolor*) and in less anthropized environments, as well as evaluate the feasibility of alternative mitigation measures where LPDs are not a viable solution. Although this study has limitations due to being a single case study, given the limited information available on the specific ecology of predators in situations of conflict with livestock and the presence of LPDs, we consider the results to be very interesting and believe they can lay the foundations for future studies that can expand our efforts.

## Figures and Tables

**Table 1:** Home range sizes (km^2^) of the LPD and ewe that were collared at a ranch in Central Argentina, during and outside lambing, calculated through the kernel method.

HR (%)	Sheep (Lambing Period)	Sheep (Outside Lambing Period)	LPD (Lambing Period)	LPD (Outside Lambing Period)
30	0.08	0.05	0.04	0.03
95	1.6	1.59	1.39	1.68
99	2.12	2.21	1.84	2.28

**Table 2:** Home range sizes and averages (km^2^) calculated using the kernel method and different percentages of locations for the foxes that were collared at a ranch in Central Argentina during and outside lambing. F: female; M: male.

Period	%	F1	F2	M1	M2	M3	M4	M5	Average
Lambing	30	-	0.86	-	0.35	0.44	0.86	0.88	0.68
	95	-	8.07	-	2.59	5.36	8.09	7.08	6.24
	99	-	11.5	-	3.59	7.99	11.38	10.3	8.96
No lambing	30	0.83	0.51	0.8	0.29	0.48	1.13	0.73	0.68
	95	8.1	4.37	7.52	3.02	4.24	10.8	5.6	6.24
	99	12.21	6.46	11.02	4.72	7.18	15.56	7.93	9.30

**Table 3:** Monthly home range sizes (calculated using the kernel method) for a LPD and a ewe that were collared at a ranch in Central Argentina, lambing months were September, October, and November.

HR (%)	2021								2022		
	September		October		November		December		January	February	March
	Sheep	LPD	Sheep	LPD	Sheep	LPD	Sheep	LPD	LPD	LPD	LPD
30	0.03	0.01	0.1	0.07	0.05	0.03	0.04	0.05	0.04	0.03	0.14
95	0.35	1.03	1.3	1.31	1.26	1.25	1.42	1.73	1.63	0.34	1.42
99	0.49	1.81	1.84	2.05	1.67	1.97	2.12	2.65	2.52	0.51	2.04

**Table 4:** Overlap (expressed as percentages) between the HRs of foxes (*n* = 7) and the HRs of the LPD and sheep that were monitored at a ranch in Central Argentina, outside the lambing period. The letters F: female; M: male.

%	LPD	Sheep	F1	F2	M1	M2	M3	M4	M5
LPD	-	75	0	11	90	0	0	2	98
Sheep	79.3	-	0	20.9	91.9	0	0	3.9	96.9
F1	0	0	-	-	-	-	-	-	-
F2	4.3	7.6	-	-	-	-	-	-	-
M1	20.2	19.4	-	-	-	-	-	-	-
M2	0	0	-	-	-	-	-	-	-
M3	0	0	-	-	-	-	-	-	-
M4	0.3	0.6	-	-	-	-	-	-	-
M5	29.5	27.5	-	-	-	-	-	-	-

**Table 5:** Bhattacharyya coefficients of overlap between the HRs of foxes (*n* = 7) and the HRs of the LPD and sheep outside the lambing period. F: female; M: male.

	LPD	Sheep	F1	F2	M1	M2	M3	M4	M5
LPD	-	0.76	0	0.03	0.43	0	0	0.003	0.49
Sheep	0.76	-	0	0.06	0.42	0	0	0.01	0.49

**Table 6:** Overlap between the HRs of foxes (*n* = 5) and the HRs of the LPD and sheep that were monitored at a ranch in Central Argentina, during the lambing period (expressed as a percentage). F: female; M: male.

%	LPD	Sheep	F2	M2	M3	M4	M5
LPD	-	90	56.2	0	10.5	0	94.9
Sheep	78.1	-	52.5	0	7.2	0	78.1
F2	9.7	10.4	-	-	-	-	-
M2	0	0	-	-	-	-	-
M3	2.7	2.1	-	-	-	-	-
M4	0	0	-	-	-	-	-
M5	18.6	21.2	-	-	-	-	-

**Table 7:** Bhattacharyya coefficients for the overlap between the home ranges (HRs) of foxes (*n* = 5) and the HRs of the LPD and sheep during the lambing period. F: female; M: male.

	LPD	Sheep	F2	M2	M3	M4	M5
LPD	-	0.86	0.15	0	0.02	0	0.41
Sheep	0.86	-	0.15	0	0.01	0	0.41

**Table 8:** Distances in meters between the centers of 30% kernel HRs of all foxes relative to the LPD and sheep in both periods. F: female; M: male.

Distance (m)	Lambing period		No Lambing Period	
	LPD	Sheep	LPD	Sheep
F1	-	-	3212.6	3115.8
F2	1719.6	1526.3	1483.9	1377.7
M1	-	-	955.8	946.7
M2	1850.1	2072.3	1602.8	1630.2
M3	2294.5	2128.9	2360.5	2204.1
M4	2160.2	2168.1	2296	2163.4
M5	1489.6	1277.2	950.1	878.9
Sheep	239.8	-	157.4	-

## Data Availability

The data that support the findings of this study are available from the corresponding author upon reasonable request.

## Abbreviations

The following abbreviations are used in this manuscript:

LPD	Livestock protection dog
HR	Home range
PEF	Patagones Experimental Farm
